# Synergistic Effects of CoHCF@MWCNTs@FA Nanocomposites Enhancing Photodynamic Therapy for Triple-Negative Breast Cancer

**DOI:** 10.1021/acsomega.5c04309

**Published:** 2026-03-12

**Authors:** Hellen C. Novais de Oliveira, Patrícia A. Matos, Tayana M. Tsubone, Edson Nossol

**Affiliations:** Institute of Chemistry, 28119 Federal University of Uberlândia, Uberlândia, MG 38400-902, Brazil

## Abstract

This study presents the synthesis of a ternary nanocomposite containing cobalt hexacyanoferrate, multiwalled carbon nanotubes, and folic acid (CoHCF@MWCNTf@FA) for application in photodynamic therapy (PDT), a minimally invasive approach for the treatment of triple-negative breast cancer. The choice of components was based on their photosensitizing properties and biocompatibility. The nanocomposites showed remarkable stability in biological medium, maintaining their structure and preventing cobalt leakage during incubation. Their efficient internalization by tumor cells (60–70%), particularly for the ternary nanocomposite, highlights the role of receptor-mediated uptake in enhancing the photodynamic response. Cell viability assays in the triple-negative breast cancer cell line (MDA-MB-231) showed that the carbon nanotube (MWCNTf) is noncytotoxic (cell viability ≥ 70%) and the isolated material CoHCF exhibited low cytotoxicity (cell viability ≥ 60%) at the tested concentrations. In contrast, under red light-emitting diode irradiation, the CoHCF@MWCNTf@FA nanocomposite induced 79% cell death at concentrations of 0.8 and 1.0 mg mL^–1^, demonstrating a significant photodynamic effect. Apoptosis data correlated with the phototoxicity results, confirming that the ternary nanocomposite showed the best performance in inducing cell death via late apoptosis of tumor cells. The ternary nanocomposite also exhibited selective phototoxicity, significantly reducing the viability of MCF-7 tumor cells (below 44%) upon irradiation, while maintaining high viability (>84%) in nontumorigenic MCF-10A cells. These results highlight the synergistic properties and potential of this nanocomposite as a promising candidate for the treatment of breast cancer through PDT.

## Graphical abstract

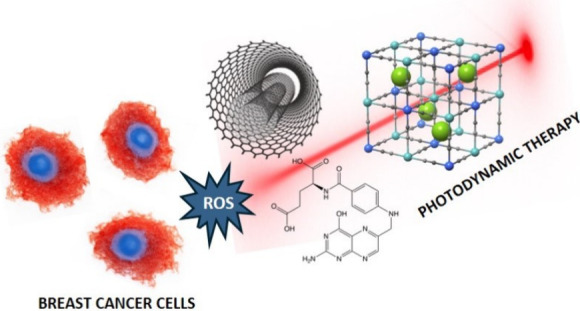

## Introduction

Breast cancer (BC) ranks as one of the most prevalent and challenging diseases in females worldwide. According to the World Health Organization (WHO), BC caused 670,000 deaths globally in 2022.^1,2^ Among the different subtypes of BC, triple-negative breast cancer (TNBC), especially the MDA-MB-231 cell line, stands out owing to its aggressiveness, high invasiveness, and metastatic nature.^3,4^ Thus, it is difficult to treat due to the lack of estrogen (ER−) and progesterone (PR−) receptors and human epidermal growth factor receptor 2 (HER2−) protein, which limits the effectiveness of hormonal and targeted therapeutic approaches. As a result, there is an urgent need to develop novel therapies for affected patients.^5,6^

In this context, photodynamic therapy (PDT) is a minimally invasive method proven to be a potential therapeutic for antitumor impacts. It is well-known that most cancer treatments have limitations, but contrasted with conventional methods like surgery, chemotherapy, and radiotherapy, PDT stands out as a hopeful approach.^7–9^ This technique specifically targets only the areas exposed to light, ensuring that healthy cells remain unaffected and minimizing the risk of collateral damage. This represents a key advantage of PDT over conventional treatments, where the lack of drug selectivity often leads to damage to healthy cells and severe side effects.^10^ PDT acts via light activation when the reaction between photosensitizers (PS) and molecular oxygen (O_2_), by using laser energy, generates cytotoxic reactive oxygen species (ROS) for killing tumor cells.^11^ Initially, the PS absorbs light and transitions from its ground state to an excited singlet state occur. This state is unstable and can decay back to the ground state, releasing energy through fluorescence or heat. However, the singlet state can also undergo intersystem crossing to a triplet excited state with a more stable and longer lifetime, which can then transfer energy to molecular oxygen, leading to the formation of singlet oxygen. This species is highly reactive and can cause oxidative damage and cell death.^12–14^

Photochemical agents are essential for the PDT process. An efficient PS must have high absorption in the visible (400–700 nm) or near-infrared (NIR) range (800–1350 nm) and be hydrophilic, nontoxic until activated, pain-free, commercially available, and biocompatible.^15^ The most extensively studied PSs (chlorins, porphyrin derivatives, and phthalocyanines) have some drawbacks such as a weak absorption band in the NIR region, compatibility, obtention, and accumulation.^16^ Nevertheless, Prussian blue (PB) and its analogs have been attracting attention as excellent candidates for PDT due to their characteristics and properties.^17^

Prussian blue (PB) is a compound that has a rich history and has been used in various fields, including art, technology, and medicine.^17^ This ancient dark blue pigment, whose chemical formula is Fe_4_^III^[Fe^II^(CN)_6_]_3_·*n*H_2_O,^18^ also named iron hexacyanoferrate (FeHCF), consists of alternating iron­(II) and iron­(III) sites octahedrally coordinated by cyanide ligands, generally generating a face-centered cubic structure.^19^ The structural arrangement of PB allows the substitution of iron sites with other transition metals, producing species similar in structure but different in composition, known as Prussian blue analogues (PBA).^20^

Apart from PB and PBAs, carbon nanotubes (CNTs) have also received increasing attention in medical fields.^21,22^ These materials are nanostructures composed of one or more curved layers of graphene^23^ that exhibit excellent thermal conductivity, optical properties, and, especially, tumor penetration ability.^24,25^ Hence, CNTs play a significant activity in targeting the tumor microenvironment (TME) by eliminating cancer cells by disrupting their basic survival conditions due to tumor penetration ability.^26,27^ Folic acid (FA) is a molecule of B-complex vitamin that has shown remarkable targeting efficiency due to its receptors, which are frequently overexpressed in several types of cancers such as uterine, ovarian, lung, and breast cancers.^28,29^ FA stands out due to its stability, affordability, and lack of immune response.^30,31^ Additionally, it exhibits a strong attraction to its receptor on the cell surface and can penetrate the cell’s interior; moreover, folate-linked drug molecules display high tumor cell specificity, offering a superior method for delivering antitumor drugs inside the cells.^32^

Additionally, the preparation of nanocomposites can significantly boost the efficacy of PDT for treating tumors. They enable improved targeting by being designed to accumulate specifically in cancer cells, minimizing damage to healthy tissues, and enhancing penetration.^33^ Furthermore, nanocomposites serve as efficient energy transducers, facilitating the conversion of light energy into ROS,^34^ which are critical for destroying tumor cells. In addition, these nanomaterials offer multifunctionality, combining roles like drug delivery and imaging enhancement.^35^ Lastly, they support combination therapies, integrating PDT with other treatments like chemotherapy to maximize therapeutic outcomes in tumor death.^36^

Hence, in this study, a nanocomposite was synthesized for the first time, under moderate conditions by a coprecipitation method and characterized by several microscopy and spectroscopy techniques. The prepared materials were sensitive to visible radiation and exhibited a notable behavior of the nanocomposites compared to the individual compounds. Moreover, CoHCF@MWCNTf@FA could be an outstanding candidate for the PDT strategy, a promising treatment against triple-negative breast cancer cells.

## Materials and Methods

### Materials

Cobalt nitrate hexahydrate (98%) and nitric acid (65%) were obtained from Synth (São Paulo, Brazil). Potassium ferricyanide­(III) (99%) and sulfuric acid (98%) were purchased from Êxodo Científica (São Paulo, Brazil). Sodium citrate dihydrate (99%) and folic acid (97%) were obtained from Panreac (Barcelona, Spain and Saint Louis, USA, respectively). Multiwalled carbon nanotubes (MWCNTs) NC7000 were acquired from Nanocyl (Belgium) with an average diameter of 9.5 nm and an average length of 1.5 μm (90%).

MDA-MB 231 cells were obtained from the American Type Culture Collection. Dulbecco’s modified Eagle medium (DMEM), 3-(4,5-dimethylthiazol-2-yl)-2,5-diphenyltetrazolium bromide (MTT), phosphate-buffered saline (PBS), and dimethyl sulfoxide (DMSO) were purchased from Sigma-Aldrich (São Paulo, Brazil). The apoptosis kits with annexin V for flow cytometry (catalog number V13245) were purchased from Thermo Fisher Scientific (USA). The 96- and 12-Transwell plates were obtained from Corning (USA). Distilled water was used for all experiments and solutions. Red (630 nm ± 180 nm; power density: 5 J cm^–2^) light-emitting LEDs were used as a light source.

### Methods

#### Instrumentation

Scanning electron microscopy (SEM) (Vega 3 TESCAN, Czech Republic) was used to investigate the surface of nanocomposites. Energy-dispersive X-ray spectroscopy (EDS) was performed using an INCA X-Act (Oxford Instruments) system attached to the SEM instrument. The crystal structure was characterized by X-ray diffractometry (XRD) (XRD6000, Shimadzu, Japan). Raman spectra were collected by a confocal Raman microspectrometer (HORIBA Scientific LabRAM HR Evolution, Horiba, Japan) using a 532 nm laser. Fourier transform infrared (FTIR) spectra were recorded on a Frontier MIR/FIR (USA) fitted with an intelligent identification of a single-lens reflex attenuated total reflection accessory (Pike Technologies, USA). The absorption spectrum was recorded on an ultraviolet–visible (UV–vis) spectrophotometer (UV-1650, Shimadzu, Japan). The flow cytometer was an Accuri C6 (Porto Alegre, Brazil).

#### Preparation of CoHCF@MWCNTs@FA and Control Materials

The synthesis procedure for functionalized MWCNTs was carried out by chemical oxidation and involved the combination of nitric and sulfuric acids. For the oxidative treatment, 0.428 g of pristine MWCNTs was mixed with 100 mL of the acid solutions (56 mL of nitric acid and 44 mL of sulfuric acid) by mechanically stirring in a hot plate for 15 min at 60 °C. Therefore, the mixture was sonicated in a conventional ultrasonic bath for 2 h, promoting CNT disentanglement within the acid solution. Then, the slurry was filtered and thoroughly washed with distilled water and ethanol until pH = 7. Finally, the slurry was dried in a convection oven at 70 °C for 24 h.

The synthesis of the nanocomposites was based on a coprecipitation method. The first step was the preparation of solutions A, B, and C. Solution A was a 50 mL mixture of 0.006 mol L^–1^ cobalt nitrate hexahydrate, 0.005 mol L^–1^ sodium citrate dihydrate, and 0.0113 mol L^–1^ folic acid. In contrast, solution B was a dispersion of 2.5 mg of functionalized MWCNTs in 100 mL of distilled water (20 min in an Ultronique 25 kHz ultrasonic bath), while solution C was a 50 mL potassium hexacyanoferrate­(III) (0.004 mol L^–1^) solution. Then, solutions were mixed by stirring for 1 h and the obtained mixture was aged at room temperature for 18 h and centrifuged with water and ethanol. Finally, the product was dried for 6 h at 80 °C. The same process was repeated for the preparation of separate CoHCF@MWCNTfs and CoHCF solids.

The preparation of the materials for the cytotoxicity assay was made by dispersing CoHCF, CoHCF@MWCNTf, and CoHCF@MWCNTfs@FA in 1 mL of Dulbecco’s modified Eagle medium (DMEM) for 15 min in 0.1, 0.2, 0.5, 0.8, and 1.0 mg mL^–1^.

#### Electrochemical Characterization

The preparation of the working electrode began with thorough washing in distilled water, followed by complete drying at 50 °C in an oven. Subsequently, 20 μL of a 1 mg mL^–1^ aqueous dispersion of CoHCF was uniformly deposited onto the glassy carbon surface using the drop-casting method, in which the material was carefully applied in a droplet form and then dried again under the same conditions. Cyclic voltammetry (CV) was performed in a three-electrode system, applying three scan cycles at a scan rate of 10 mV s^–1^ within a potential range of 0.0–1.0 V relative to the Ag(s)/AgCl(s)/Cl^–^(sat.) reference electrode and using a platinum wire as the counter electrode. The solution contained 0.1 mol L^–1^ KCl as the supporting electrolyte. This procedure was fully replicated for the CoHCF@MWCNTf and CoHCF@MWCNTf@FA nanocomposites.

#### Cell Culture and Treatments

The human breast cancer cell lines, MDA-MB-231 and MCF-7, were grown in Dulbecco’s modified Eagle medium (DMEM) supplemented with 10% (v/v) fetal bovine serum (FBS), 100 μg/mL penicillin, and 100 μg mL^–1^ streptomycin, while MCF-10A cells were cultured in medium supplemented with 5% (v/v) fetal bovine serum (FBS), 1% (v/v) penicillin, 100 ng/mL toxin, 0.5 μg/mL hydrocortisone, 20 ng/mL epidermal growth factor (EGF), and 10 μg/mL insulin. The cells were maintained in an incubator in 5% CO_2_ at 37 °C. The cells were removed by trypsin and washed with phosphate-buffered saline (PBS) for the experiments. The sterilization of the nanocomposites was performed using the autoclaving method. Samples were weighed at concentrations of 0.1, 0.2, 0.5, 0.8, and 1.0 mg mL^–1^, transferred into amber glass tubes, and autoclaved at 121 °C for 40 min. To further ensure the removal of potential endotoxin contaminants, the samples were subsequently incubated in a drying oven at 60 °C for 24 h. Following sterilization, the nanocomposites were resuspended in 1 mL of DMEM culture medium and dispersed for 20 min to ensure homogeneity.

#### Cell Viability Assay via the MTT Method

One of the plates was considered a control plate (no LED irradiation), and the other plate was irradiated. The reference plate was kept without light incidence for 15 min, and the irradiated one was under a red LED for the same time. The photodynamic assay was made by the conditions shown in Table S1. The power in mW of the red LED was previously measured with an optical power meter Newport model 19M6-R connected to a detector Newport model 818-UV S/N 10488 (USA).

The cytotoxicity was measured after 24 h of incubation of both radiated and irradiated plates. The cell viability was tested by the colorimetric assay using a 3-(4,5-dimethylthiazol-2-yl)-2,5-diphenyltetrazolium bromide (MTT) assay to determine the cell survival. This process can be analyzed as a mitochondrial reductase mechanism. Living cells convert the MTT compound to an insoluble formazan crystal. The resulting formazan was solubilized using DMSO, and its concentration was determined using spectrophotometric methods. Briefly, in the culture medium was added 80 μL of MTT (0.75 mg mL^–1^) under 5% CO_2_, for 3 h at 37 °C. The resulting purple formazan crystals were dissolved in 100 μL of DMSO. Then, the absorbance at 570, 630, and 690 nm was recorded using a microplate reader. These experiments were carried out for all the cell lines involved.

#### Cellular Uptake Analysis

MDA-MB-231 cells were seeded in 12-well plates at a density of 1.5 × 10^5^ cells per well in 1 mL of DMEM and incubated for 18–24 h at 37 °C and 5% CO_2_ to allow adhesion. Stock solutions of the nanocomposites were prepared in DMEM and diluted to the working concentration of 0.5 mg/mL prior to incubation. Cells were then incubated with the nanocomposites for 4 h under standard culture conditions. After incubation, 1 mL of the supernatant was collected from each well and mixed with 1 mL of 10% Triton X-100 or 10% SDS for absorbance measurements (200–800 nm) using a microplate reader. The adherent cells were washed with PBS, lysed with 1 mL of 10% Triton X-100 or 10% SDS, and supplemented with 1 mL of DMEM to ensure consistent dilution. The lysates were homogenized, and their absorbance spectra (200–800 nm) were also recorded to determine the amount of nanocomposite internalized by the cells.

#### In Vitro Cell Apoptosis Detection Assay

The preparation of the materials for the apoptosis assay was similar to the cytotoxicity and phototoxicity assay. The main distinctions were selecting a single concentration (1 mg mL^–1^) for dispersing materials and culturing MDA-MB-231 cells using a 12-well plate with 1 × 10^6^ cells/well. Treated and control cells were detached through trypsinization, washed and centrifuged twice with PBS, and resuspended in 625 μL of binding buffer. 5 μL of annexin V-FITC and 5 μL of propidium iodide (final volume of 500 μL to each sample) were added, followed by incubation at room temperature in the dark for 15 min. Finally, fluorescence emission was analyzed by cytofluorometry in an Accuri C6 flow cytometer. For annexin V-FITC, excitation was at 488 nm with emission at 530 ± 30 nm (FL1), and for propidium iodide (PI), excitation was at 633 nm and emission at 670 nm long pass (FL3). Samples were analyzed in triplicate, with at least 20,000 events collected per analysis. The data obtained were processed and analyzed using FlowJo software.

## Results and Discussion

### Scanning Electron Microscopy (SEM)

The SEM images revealed the morphological characteristics of the materials composed of CoHCF, CoHCF@MWCNTf, and CoHCF@MWCNTf@FA, as shown in Figure 1. In Figure 1a, an agglomerated nanocube structure of CoHCF was observed. In Figure 1b, it was noted that the MWCNTs were fully intertwined with the CoHCF nanocubes, forming an integrated network in the CoHCF@MWCNTf nanocomposite. The presence of FA was not detected in the SEM images (Figure 1c), likely due to its low content and high dispersion. These results indicate that the MWCNTs significantly influenced the size of the CoHCF nanomaterials, as can be observed in the histograms (Figure 1g–i) presented alongside the SEM images. For a more precise evaluation of the size and morphology of the synthesized materials, all samples were analyzed by TEM. As evidenced in the images in Figure 1d–f, the nanocomposites revealed the presence of CoHCF nanoparticles with a well-defined morphology in all compounds, distributed among the carbon nanotubes, which exhibit a "spaghetti-like" structure. Additionally, especially in the nanocomposites containing MWCNTs, the presence of smaller-diameter nanocubes at the vertices of the larger-diameter cubes was observed.

**Figure 1:**
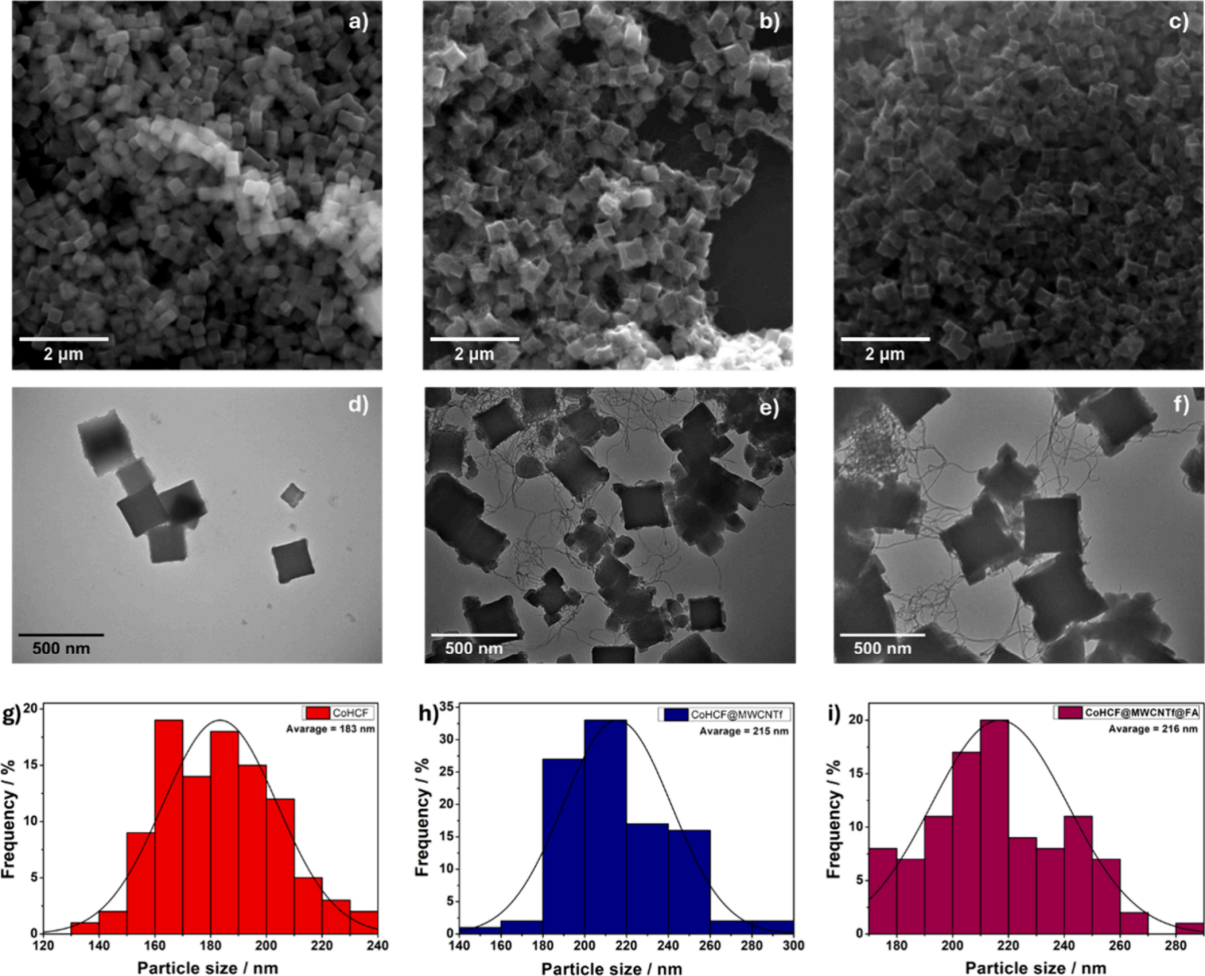
SEM and TEM images of CoHCF (a, d), CoHCF@MWCNTf (b, e), and CoHCF@MWCNTf@FA (c, f) and their respective histograms (g–i).

### Energy-Dispersive X-ray Spectroscopy (EDS)

The elemental composition of the synthesized materials is presented in Figure S1. For this characterization, all nanomaterials were deposited on silicon (Si) substrates, which explains the presence of this element in all spectra. The presence of potassium (K) is attributed to the use of potassium ferricyanide K_3_[Fe­(CN)_6_], which occupies the interstitial sites of the hexacyanoferrate structure. The spectrum of CoHCF revealed characteristic peaks for the elements iron (Fe), cobalt (Co), nitrogen (N), and carbon (C), confirming the elemental composition of CoHCF. In the samples corresponding to the nanocomposites, the same basic elemental composition was observed, with a notable increase in the Co/Fe ratio. This Co/Fe ratio compared with the pure material is 1.38, while for the CoHCF@MWCNTf and CoHCF@MWCNTf@FA nanocomposites, ratios of 1.40 and 1.46 were observed, respectively. This increase can be attributed to a decrease in Fe content, suggesting a higher number of ferricyanide vacancies, indicating that the MWCNTfs and FA induce the formation of these structural defects.

### X-ray Diffraction (XRD)

The X-ray diffraction technique was employed to investigate the crystalline structure of the materials. Figure 2a presents the diffractograms of the synthesized materials.

**Figure 2:**
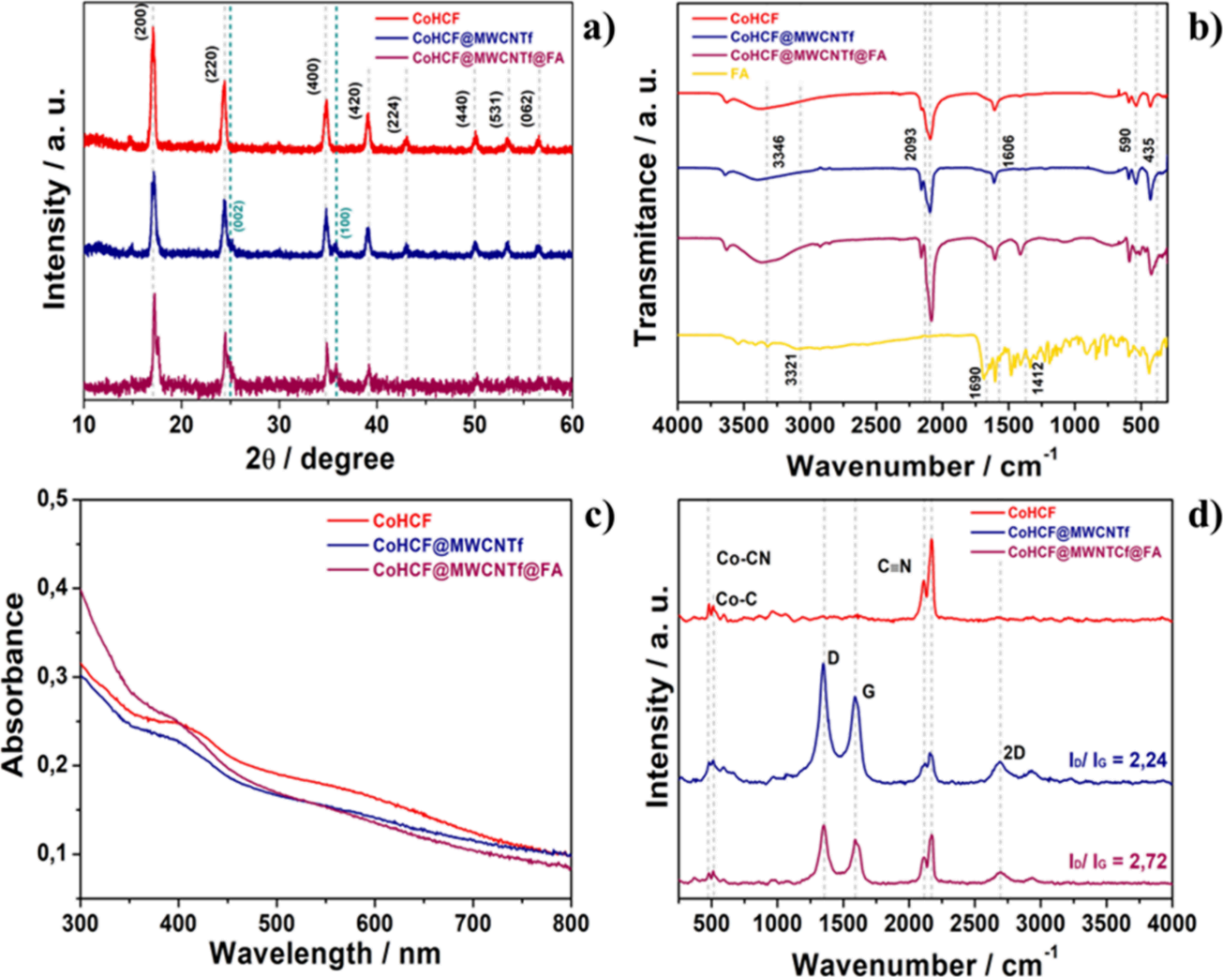
Diffractograms (a), FTIR spectra (b), UV–vis, (c) and Raman spectra (d) of CoHCF, CoHCF@MWCNTf, and CoHCF@MWCNTf@FA. Panel (b) also shows the FA spectrum for comparison.

For all samples (Figure 2a), diffraction peaks corresponding to the face-centered cubic (fcc) crystalline structure of CoHCF were observed, associated with the *hkl* planes (200), (220), (400), (420), (224), (440), (531), and (620), indexed according to the JCPDS crystallographic file (JCPDS 86-0502).^37^ Additionally, peaks were observed in the region of 2θ = 25° and 36° in the CoHCF@MWCNTf and CoHCF@MWCNTf@FA samples, which can be associated with the interplanar distance of the carbon nanotube walls and the alignment and regularity of the hexagonal plane, corresponding to the (002) and (100) planes, respectively. The peaks related to the carbon nanotubes suggest that the crystalline lattice structure of the carbon material was not damaged by functionalization.^38^

A slight shift of the peaks to higher 2θ values was also observed in the diffractograms of the nanocomposites compared to pure CoHCF, suggesting lattice modification. This phenomenon can be attributed to the presence of MWCNTfs and FA in the hexacyanoferrate structure, causing a change in the unit cell parameters of the hexacyanoferrate and the formation of [Fe­(CN)_6_] vacancies.^39^ Additionally, Figure S2 shows an additional peak at 17.7° for CoHCF@MWCNTf@FA, indicating the distortion of the cubic hexacyanoferrate lattice into a rhombohedral phase, suggesting a variation in the amount of K^+^ in the lattice that alters its structure.^40–42^

### Fourier Transform Infrared Spectroscopy (FTIR)

The materials prepared in this work were also characterized by infrared absorption spectroscopy. Figure 2b reveals that in all the compounds analyzed containing CoHCF, the bands υ­(OH), δ­(HOH), and υ­(CN) were identified approximately at 3346, 1606, and 2093 cm^–1^, corresponding to hydroxyl groups, the presence of water, and cyanides, respectively, as shown in Table S2. The υ­(OH) band can provide information about the coordination of the water molecule. Its observation in the absorption range of 3000–3700 cm^–1^ is a clear indication of the presence of −OH groups in water molecules within the material’s structure. This feature is typical of water molecules embedded in the structural cavities, but not forming strong bonds with the material’s lattice. Additionally, the band at 3346 cm^–1^ is attributed to water molecules strongly bonded by hydrogen bonds in the structure, as reported in a previous study.^43^ This band indicates that, in addition to the weakly bonded water molecules, there is also a fraction of water molecules that are firmly coordinated to the lattice atoms, likely occupying critical positions in the structure, such as the coordination sites of metal cations.^44^

The bands presented at wavenumbers below 600 cm^–1^ are related to the vibrations of M–NC bonds. The nanocomposites and control materials showed bands in the 422 and 590–537 cm^–1^ region, associated with δ­(Co–NC–Fe) and υ­(Fe–C), respectively.^45^

In Figure S3, it is highlighted that the relative intensity of the C≡N stretching, related to the FeII–CN–CoII, increases and the position has shifted to lower wavenumbers, indicating a greater formation of the fully reduced HCFCo species, as well as a possible interaction between the analog and FA. However, the formation of FeIII–CN–CoII mixed with small amounts of FeII–CN–CoIII can also be observed, suggesting the presence of a mixture of different oxidation states of Fe and Co within the CoHCF structure.^46^

The isolated FA spectrum displayed characteristic vibrations of ν­(NH), ν­(CO), and δ­(CH), which were also identified in the spectrum of HCFCo@NTCf@AF, confirming the presence of FA in the ternary nanocomposite. The bands at 3321 cm^–1^ correspond to amine groups, at 1412 cm^–1^ are attributed to C–H groups, and at 1690 cm^–1^ are related to the free ketone of the carboxyl group.^47^ These observations confirm the successful incorporation of FA into the CoHCF@MWCNTf@FA nanocomposite structure.

### UV–Vis Spectroscopy

In Figure 2c, the dispersion of CoHCF and their composites in water was investigated by UV–vis spectroscopy in the 300–800 nm range using the concentration of 0.05 mg mL^–1^. HCFs exhibit distinct spectral characteristics that reflect their complex electronic interactions and spin states. In the absorption spectrum of CoHCF and its nanocomposites, a band around 400–420 nm is observed, attributed to the ligand-to-metal charge transfer (LMCT).^46,48^ Additionally, a band between 500 and 700 nm is observed, corresponding to the intervalence charge transfer between metal ions. These electronic transitions are indicative of the photochemical and photophysical properties of the HCFs, reinforcing their potential for applications in photodynamic therapies and other uses based on their visible-light absorption and charge transfer capabilities.^49,50^ On the other hand, bands related to MWCNTfs are not observed in the spectrum, as their absorption predominantly occurs in the UV region, around 250 nm. This behavior suggests that, although the nanotubes are present in the nanocomposite structure, their spectral contribution is not perceptible in the visible region.^51^ The spectra of CoHCF@MWCNTf and CoHCF@MWCNTf@FA showed similar signals, with absorbance values lower than those of pure CoHCF. This reduction can be attributed to the presence of MWCNTf in the nanocomposites, which have a high light absorption capacity and multiple reflections within the nanotube cavities, contributing to the decrease in overall absorbance.^52^

### Raman Spectroscopy

The Raman spectra of the materials were collected using a laser with a wavelength of 532 nm, represented in Figure 2d. In all the spectra, bands around 2100 cm^–1^ are observed, attributed to the vibrational modes of the CN groups. Wavenumber values below 600 cm^–1^, specifically the broad bands at 468–520 cm^–1^, are associated with the stretching modes of Fe–C and Co–N, as well as the deformation of FeCo–NC.^53^ The nanocomposites displayed three distinct Raman-active bands attributed to MWCNTf, centered at 1347, 1591, and 2692 cm^–1^, representing the D, G, and 2D bands of the carbon structure, respectively.

The D band requires structural defects for activation and indicates the presence of heteroatoms, sp^3^-hybridized carbons, and other defects.^54^ The G band generally appears in all sp^2^ materials and is related to the stretching mode of the C=C bonds in the hexagonal lattice of carbonaceous materials.^55^ The 2D band does not require defects for activation and typically appears around 2600–2700 cm^–1^; its shape and position are important parameters indicating the number of graphene layers and their quality.^56^

The intensity ratio of the D and G bands (ID/IG) serves as a parameter to determine the degree of disorder in the MWCNTf structures. A high ID/IG ratio is indicative of an increase in the number of defects present in the materials. Spectral analyses of functionalized MWCNTf revealed an ID/IG ratio of 2.07, while CoHCF@MWCNTf and CoHCF@MWCNTf@FA exhibited ratios of 2.24 and 2.72, respectively. These observations suggest that the synthesis process of the nanocomposites, in the presence of FA, led to structural modifications and an increase in the defect density in the MWCNTfs.

### Thermogravimetric Analysis

The thermal stability, temperature range, and decomposition behavior of the materials are shown in Figure 3.

**Figure 3:**
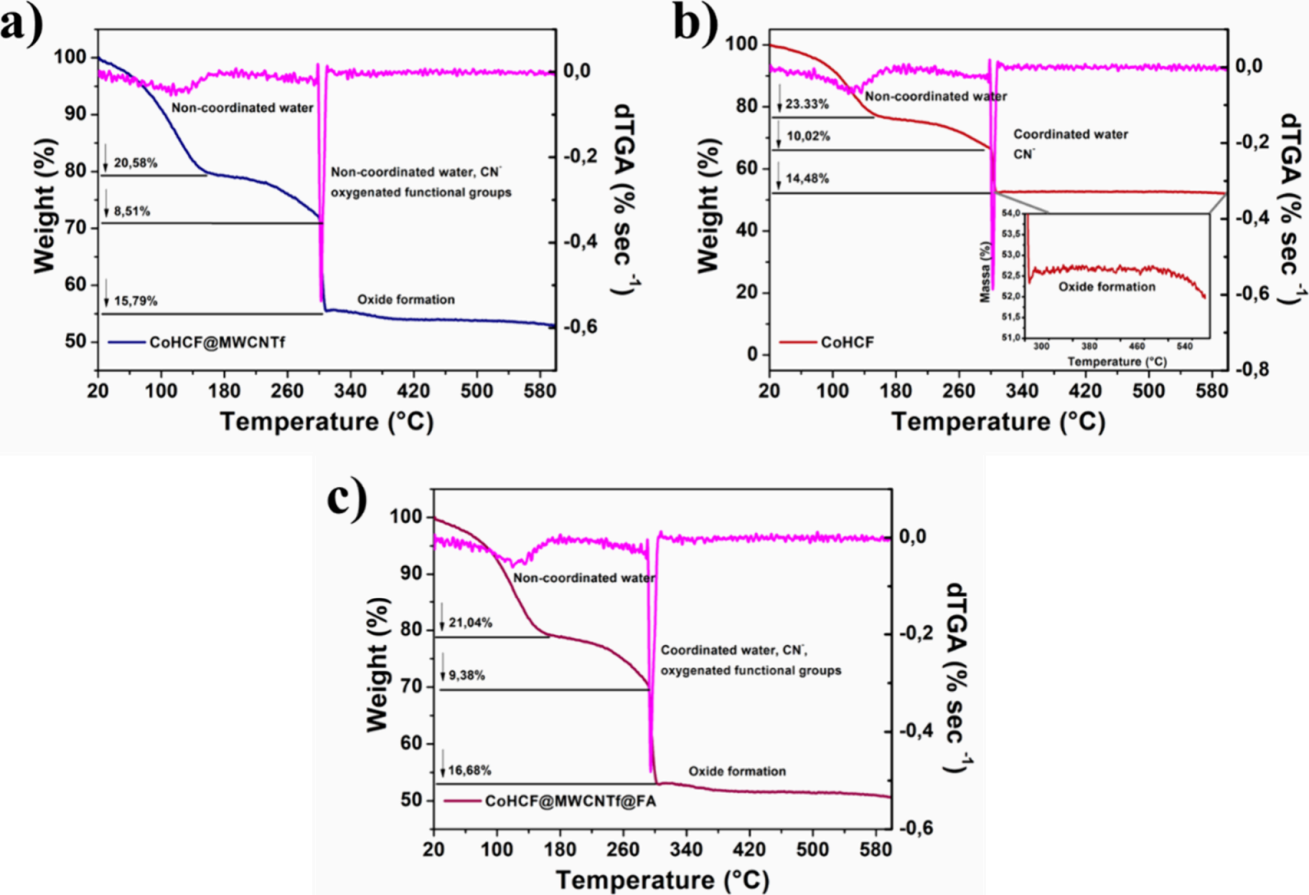
Thermogravimetric analysis of CoHCF (a), CoHCF@MWCNTf (b), and CoHCF@MWCNTf@FA (c).

As discussed above, two types of water molecules are present in the AAP structure: weakly bound water molecules located in structural cavities and strongly coordinated water molecules occupying positions in the crystalline lattice. The removal of noncoordinated water molecules occurs in the 80–150 °C range for all materials, resulting in a mass loss of approximately 23% for CoHCF and 21% and 20% for the CoHCF@MWCNTf@FA and CoHCF@MWCNTf nanocomposites, respectively. This result indicates that the presence of nanotubes makes the sample slightly less hygroscopic than the pure sample.

The removal of coordinated water molecules occurs between 160–300 °C, with a total mass loss of around 45% for all materials.^37,57,58^ Beyond this temperature range, the elimination of CN^–^ groups is observed, along with the oxygenated functions of carbon nanostructures in the nanocomposites and functional groups of the FA molecule. Above 300 °C, mass variations can be attributed to oxide formation. The temperature derivatives shown in Table S3 were obtained from the TGA curves to identify the point of highest mass loss rate. The dTGA analysis shows that the first decomposition, associated with the loss of adsorbed water molecules, occurs between 119–125 °C and is influenced by MWCNTf and FA. The incorporation of MWCNTf reduces the decomposition temperature to 119 °C and the mass loss to 20.58% due to its hydrophobicity, while the addition of FA increases this temperature to 125 °C, suggesting greater water molecule retention.

The second decomposition (270–278 °C) is related to the degradation of cyano groups and organic matter, shifting to higher temperatures as materials are incorporated into the analog structure, indicating increased thermal stability. Pure CoHCF decomposes at 270 °C, whereas CoHCF@MWCNTf and CoHCF@MWCNTf@FA show higher values (272 and 278 °C, respectively), with lower mass losses, suggesting interactions between the materials.

The third decomposition (295–302 °C) is associated with the final degradation of the matrix, remaining at 302 °C for CoHCF and CoHCF@MWCNTf, but decreasing to 295 °C in CoHCF@MWCNTf@FA, possibly due to the thermal degradation of FA. This also results in the highest mass loss (16.68%), suggesting that its incorporation alters the system’s stability.

### Electrochemical Study–Cyclic Voltammetry

PDT relies on the generation of reactive oxygen species (ROS), particularly singlet oxygen (^1^O_2_), upon light activation of a photosensitizer, and the redox behavior of such compounds directly influences their therapeutic efficacy. CV allows the prediction of the ease with which a material can donate or accept electrons, which correlates with its ability to interact with molecular oxygen and generate ROS upon light activation. Figure 4 shows the cyclic voltammogram of the electrodes at a scan rate of 10 mV s^–1^ within a potential window of 0.0–1.0 V relative to an Ag(s)/AgCl(s)/Cl^–^(sat.) electrode, with 0.1 mol L^–1^ KCl as the supporting electrolyte.

**Figure 4:**
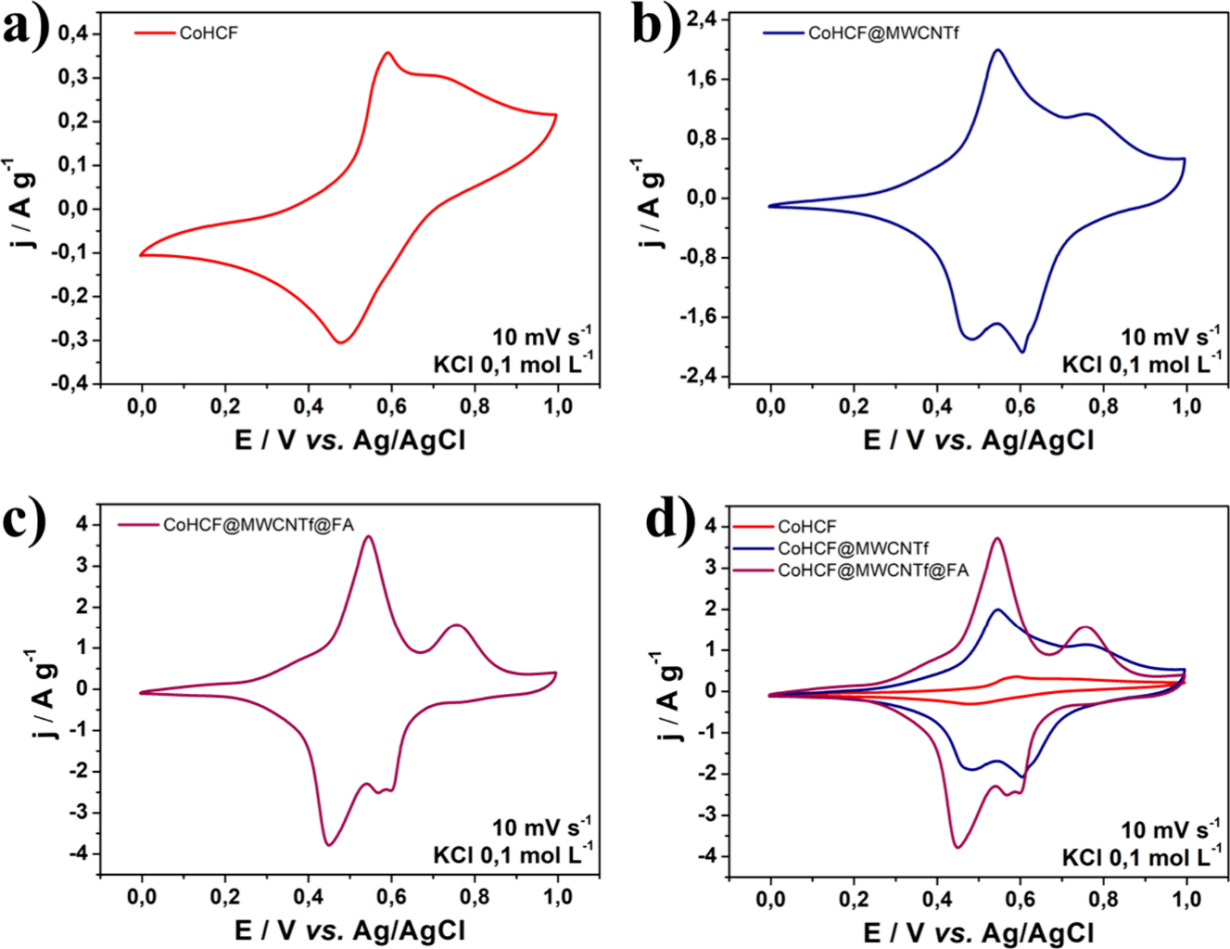
Cyclic voltammogram of CoHCF (a), CoHCF@MWCNTf (b), CoHCF@MWCNTf@FA (c), and the plot of all materials (d).

In Figure 4a, two well-defined redox peaks are located at approximately 0.5 V. Peak I corresponds to the Fe­(II)/Fe­(III) redox reaction in CoHCF, accompanied by the insertion and extraction of K^+^ ions within the metallic framework to maintain local charge neutrality. Peak II at 0.7 V is attributed to Co­(II)/Co­(III) transitions.^59,60^

A significant increase in current is observed in Figure 4b, indicating the influence of MWCNTf, whose electronic properties greatly enhance electron transfer due to their large specific surface area and high conductivity. Additionally, a slight shift in the peak potentials of the Fe­(II)/Fe­(III) and Co­(II)/Co­(III) redox pairs is observed, reflecting changes in reaction dynamics due to the structural modification introduced by MWCNTf.

Figure 4c, corresponding to CoHCF@MWCNTf@FA, exhibited similar results to the CoHCF@MWCNTf nanocomposite, regarding the presence of redox pairs derived from HCFCo and the characteristic voltammetric influence of MWCNTf. Furthermore, a splitting of the cathodic peak at 0.6 V was noted in materials containing MWCNTf, which may be associated with the mixture of cobalt oxidation states in different chemical environments.^61–64^ High positive *E*_ox_ values indicate that the compound has difficulty undergoing oxidation, which may compromise the efficiency of ROS generation via type I mechanisms based on electron transfer. Conversely, low negative *E*_red_ values suggest that the compound is difficult to reduce, which may limit its involvement in the reductive pathway of ROS generation. The potentials obtained for the materials prepared in this work (between 0.4 and 0.8 V) are in the ideal range for ROS generation compared with other photosensitizers used in PDT, such as cobalt-phthalocyanines (−0.36/+0.44 V).^65^

### Stability Studies in Biological Media

The stability of the nanocomposites in DMEM was evaluated by UV–vis spectroscopy over 24 h of incubation (Figure S4). For all samples, including CoHCF, CoHCF@MWCNTf, and CoHCF@MWCNTf@FA, the characteristic absorption band at approximately 500–550 nm, attributed to the Co–Fe charge transfer transition, was preserved throughout the analysis, indicating that the Prussian blue analogue framework remained preserved.

Compared to the initial spectrum (0 h), all materials exhibited a gradual increase in absorbance intensity up to around 8–12 h, followed by stabilization at 24 h. This behavior suggests a progressive improvement in dispersion within the biological medium rather than structural degradation or cobalt leaching. The incorporation of MWCNTs and subsequent with FA did not significantly alter the spectral profile, confirming that these surface modifications maintain the optical and structural stability of the nanocomposites in DMEM. Overall, these results demonstrate that the designed materials exhibit high stability under physiological-like conditions, supporting their suitability for biological and cellular studies.

### PDT Effect of CoHCF@MWCNTf@FA on MDA-MB-231 Cells

The effectiveness of the PDT of the nanocomposites was investigated by evaluating their phototoxicity in MDA-MB-231 cells under red light exposure (λ_max_ = 630 nm, light dose = 5 J cm^–2^) with nanocomposite concentrations ranging from 0.1 to 1.0 mg mL^–1^ (Figure 5). To analyze whether the toxicity in the dark of nanocomposites was negligible or not, cell viability of the samples was also performed in the absence of light (in the dark).

**Figure 5:**
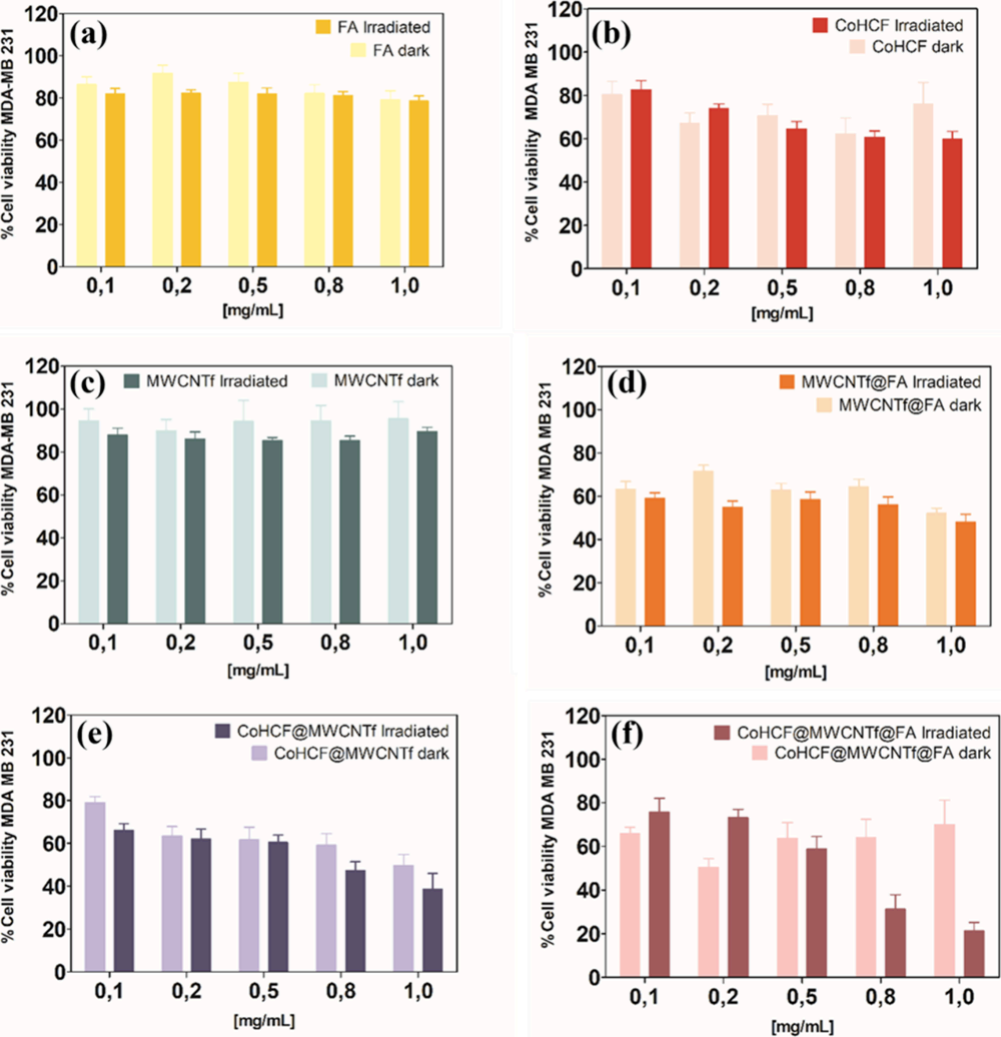
Studies of the (%) viability of MDA-MB-231 cells treated with FA (a), CoHCF (b), MWCNTf (c), MWCNTf@FA (d), CoHCF@MWCNTf (e), and CoHCF@MWCNTf@FA (f) at different concentrations (0.1–1.0 mg mL^–1^) under red LED irradiation (λ_max_ = 630 nm, light dose = 5 J cm^–2^) or protected from light (dark). The bars represent the mean ± standard deviation of 5 independent experiments, with 4 repetitions in each replicate (*n* = 20) for dark tests, and independent duplicates, with 4 repetitions in each replicate (*n* = 8) performed in the presence of light. Statistical analysis was performed using one-way ANOVA followed by Tukey’s multiple-comparison test, with statistical significance set at *p* ≤ 0.05.

It is observed that cells treated only with FA (Figure 5a) or MWCNTf (Figure 5c) remained viable (cell viability remained close to 100%) across all concentrations used (0.1–1.0 mg mL^–1^). This indicates that FA or MWCNTf, when used alone, is noncytotoxic (cell viability ≥ 70% of the control group), either in the dark or under radiation (Figure 5a,c). The MWCNTf@FA nanocomposite exhibited around 40% cytotoxicity at almost all concentrations used (Figure 5d). However, in the presence of light, MWCNTf@FA did not increase the toxicity, indicating the absence of a photodynamic effect (Figure 5d).

The CoHCF compound exhibited approximately 30% toxicity to MDA-MB-231 cells, depending on the concentration used (Figure 5b). However, no phototoxicity was observed for CoHCF under red LED irradiation at most concentrations studied, as there was no significant difference in cell viability between conditions in the dark and under irradiation (Figure 5b), except for the highest concentration of 1.0 mg mL^–1^. The cytotoxicity of the CoHCF@MWCNTf nanocomposite remained around 30% for MDA-MB-231 cells between 0.1 and 0.5 mg mL^–1^ and was independent of light exposure (Figure 5e). This result shows that when CoHCF is associated with MWCNTf, it does not significantly increase the toxicity already present when CoHCF is used alone (Figure 5e).

The CoHCF@MWCNTf@FA nanocomposite also exhibited around 30% toxicity in the dark (Figure 5f), which is attributed to the toxicity of CoHCF alone, as observed when used independently (Figure 5b), and maintained in the CoHCF@MWCNTf nanocomposite (Figure 5e). Although the CoHCF@MWCNTf@FA nanocomposite induced some reduction in cell viability under dark conditions—reaching approximately 30% cell death at the highest concentration tested (1.0 mg mL^–1^)—this effect remains within an acceptable range for preliminary *in vitro* studies involving nanomaterials with antitumor applications. Given that triple-negative breast cancer is highly aggressive and resistant to conventional therapies, a moderate level of dark toxicity may even contribute positively to the therapeutic efficacy of the treatment. In contrast to other nanocomposites, for CoHCF@MWCNTf@FA, a significant difference was observed in the cell viability percentage between the dark and light conditions, especially at higher concentrations. It was observed that, on average, 79% of MDA-MB-231 cells died in contact with the nanocomposite at the concentration of 1.0 mg mL^–1^ under light exposure.

To compare the photodynamic performance of the synthesized nanocomposite with conventional photosensitizers, the cytotoxic and phototoxic profiles of methylene blue (MB) and Azure A were evaluated under identical experimental conditions (λ = 630 nm, light dose = 5 J cm^–2^). The comparative cell viability results for MDA-MB-231 cells are shown in Figure 6. Under red light irradiation, methylene blue (MB) exhibited a clear concentration-dependent phototoxic response, reducing cell viability from ∼80% at 2.39 mg mL^–1^, whereas dark controls remained above 75% viability across all concentrations. A similar behavior was observed for Azure A, which decreased cell viability to ∼80% at 2.19 mg mL^–1^ under irradiation, while maintaining viability above 70–80% in the dark. This result demonstrates that the CoHCF@MWCNTf@FA nanocomposite achieves an equivalent or superior photodynamic response using less than half the photosensitizer concentration required for methylene blue (MB) or Azure A to reach similar efficacy.

**Figure 6:**
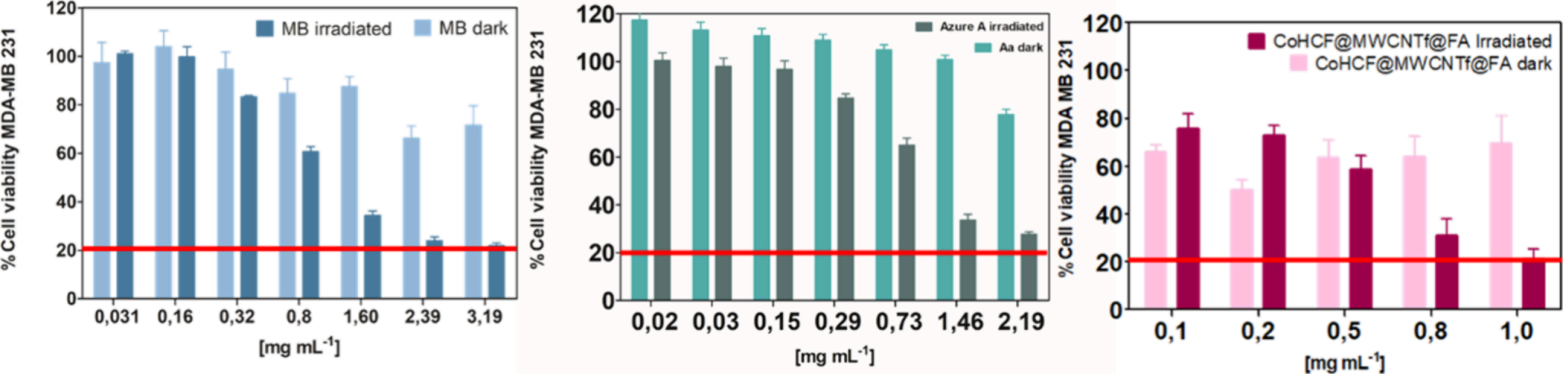
Percentage of cell viability of methylene blue (MB), Azure A, and CoHCF@MWCNTf@FA nanocomposite under red light exposure (λ = 630 nm, light dose = 5 J cm^–2^).

These findings indicate a higher photodynamic efficacy of the CoHCF@MWCNTf@FA system compared to conventional photosensitizers (methylene blue and Azure A), reinforcing the nanocomposite’s potential as a next-generation multifunctional PDT platform.

To further evaluate the selective cytotoxicity of the CoHCF@MWCNTf@FA nanocomposite, its effects were investigated on both cancerous and noncancerous cell lines, aiming to assess its specificity and safety profile. As evidenced by the data presented in Figure 7, the nanocomposite CoHCF@MWCNTf@FA exhibited a pronounced and selective phototoxic effect upon light activation. In MCF-7 breast cancer cells (Figure 7a), the percentage of viable cells decreased significantly in a concentration-dependent manner after irradiation, with viability dropping below 44% at concentrations of 0.8 and 1.0 mg mL^–1^. This clearly demonstrates the nanocomposite’s ability to induce cytotoxicity through a photodynamically triggered mechanism. In the absence of light, however, MCF-7 cell viability remained high, with more than 94% of cells alive at concentrations up to 0.8 mg mL^–1^, indicating low basal cytotoxicity without irradiation.

**Figure 7:**
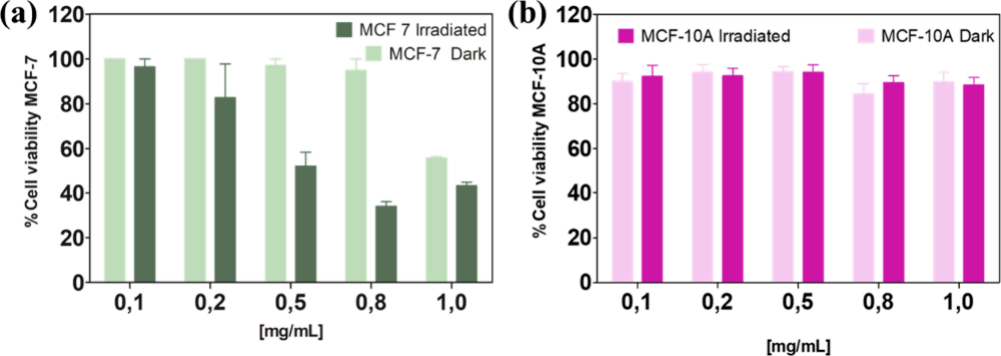
Studies of the (%) viability of tumoral cells MCF-7 (a) and nontumoral cells MCF-10A (b) treated with CoHCF@MWCNTf@FA at different concentrations (0.1–1.0 mg mL^–1^) under red LED irradiation (λ_max_ = 630 nm, light dose = 5 J cm^–2^) or protected from light (dark). The bars represent the mean ± standard deviation of 2 independent experiments, with 4 repetitions in each replicate (*n* = 8) for dark and irradiated tests.

Remarkably, the nanocomposite does not exhibit cytotoxicity to nontumorigenic MCF-10A cells (Figure 7b). Regardless of the presence or absence of light, over 84% of MCF-10A cells remained viable across all tested concentrations, highlighting minimal toxicity to healthy cells. This selective cytotoxic profile between cancerous and healthy cells underscores the potential of CoHCF@MWCNTf@FA as an effective and safe photosensitizer for localized photodynamic therapy. Overall, these results reinforce the nanocomposite’s promise for targeted breast cancer treatment, combining strong photodynamic efficacy with minimal off-target effects in normal tissues.

### Flow Cytometry with Dual Labeling of Annexin V and Propidium Iodide (FITC/PI)

For the analysis about exposing annexin V and/or internalization of PI during the cell death process, we performed flow cytometry using the markers annexin V-FITC and PI, as shown in Figure 8, and provided important data for understanding the pathway of cell death involved after PDT on MDA-MB-231 cells.

**Figure 8:**
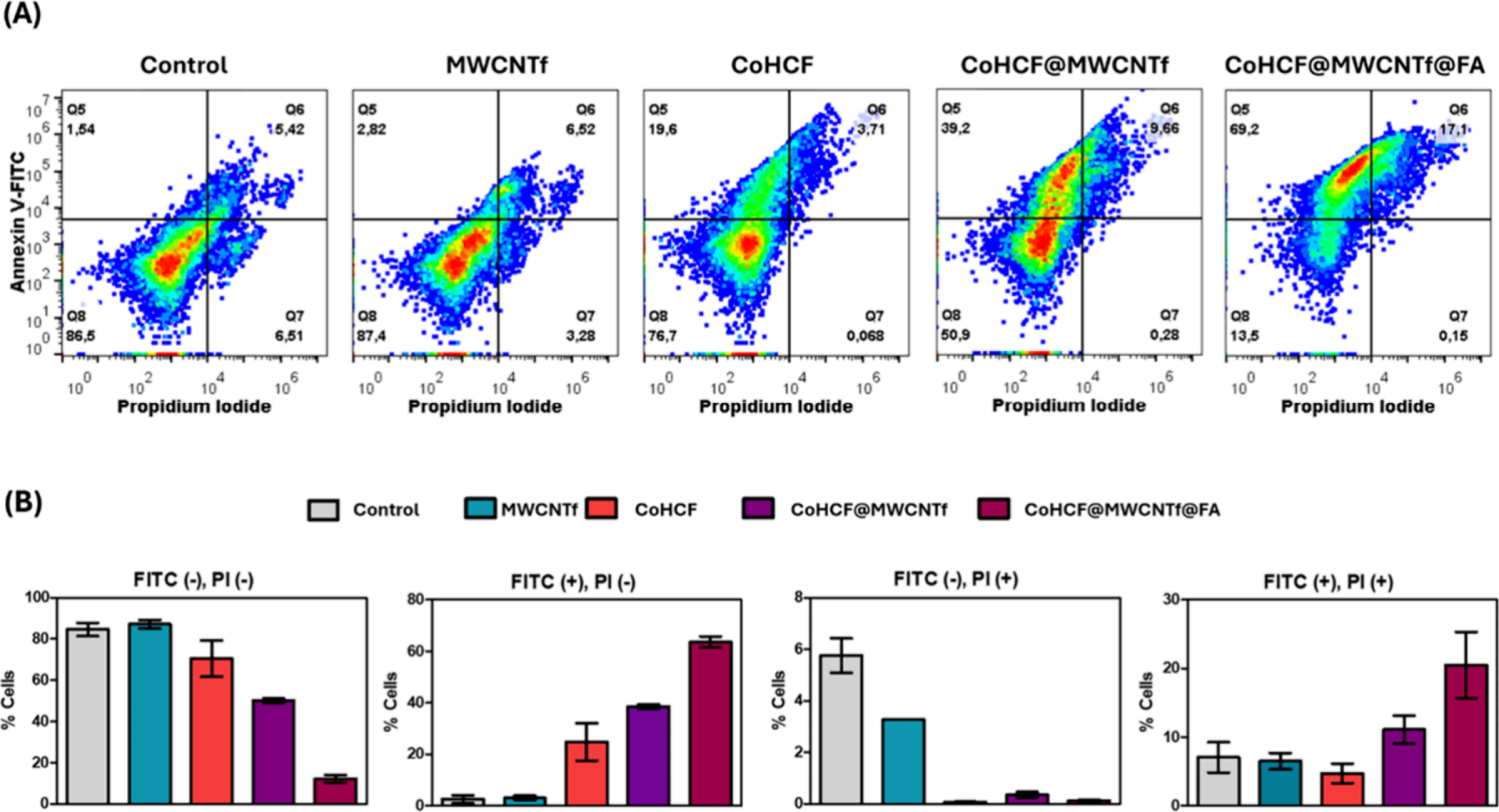
Pseudocolor scatter plots showing the definition of 4 populations, according to the positive and negative responses to annexin V-FITC and propidium iodide, in untreated cells (control) and cells treated with 1 mg/mL of MWCNTf, CoHCF, CoHCF@MWCNTf, and CoHCF@MWCNTf@FA and irradiated with red LED (λ_max_ = 630 nm, light dose = 5 J/cm^2^) (a). Bar graphs with the percentages of each quadrant highlight the relative change compared to the control of the 4 defined populations (b).

The untreated cells and those treated with MWCNTf showed a predominance of viable cells (≈ 87%, Figure 8), as evidenced by the FITC(−), and PI(−) labeling. Additionally, the incidence of cells labeled with annexin V only (FITC­(+), PI(−)), was low, suggesting that MWCNTf did not induce significant phototoxic effects. This result aligns with the cell viability data, which show that MWCNTs are nontoxic materials (in the presence or absence of light).

In contrast, treatment with CoHCF resulted in moderate induction of early apoptosis (19.6% FITC+/PI–, Figure 8), evidenced by an increase in the proportion of cells labeled only with annexin V. There was no significant number of cells with double labeling (3.71% FITC+/PI+) or labeled only with PI (0.07% FITC–/PI+). CoHCF showed approximately 20% toxicity in the cell viability data by the MTT method (Figure 5b).

When CoHCF was combined with MWCNTf to form the nanocomposite CoHCF@MWCNTf, there was an increase in the percentage of cells exposing annexin V to the external membrane (39.2% FITC+/PI–, Figure 8) and cells exposed to both probes (9.66% FITC+/PI+, Figure 8) compared to CoHCF alone. This suggests that the combination of MWCNTf with CoHCF enhanced a cell death type involving annexin V exposition when exposed to light.

The nanocomposite CoHCF@MWCNTf@FA resulted in an increased rate of early apoptosis (69.2% FITC+/PI–, Figure 8) and late apoptosis or necrosis (17.1% FITC+/PI+, Figure 8). This increase in apoptosis suggested that the combination of CoHCF with MWCNTf and FA potentiated the phototoxic effects of these materials, creating a more favorable environment for inducing programmed cell death. The hypothesis that bioactive molecules, such as FA, played a crucial role in improving the therapeutic efficacy of the materials was reinforced by results showing that these molecules facilitated the internalization of the nanomaterials into tumor cells, promoting a more efficient interaction between the compounds and their target cells.

Thus, these results revealed that the materials have considerable potential as an antitumor agent, as annexin V/PI data align with phototoxicity data via MTT assay, as shown in the viability graphs in Figure 5, highlighting the potential of the CoHCF@MWCNTf@FA nanocomposite as a proposal to optimize therapeutic photodynamic effects.

### Cellular Uptake Analysis

To further understand the enhanced photodynamic performance of the ternary nanocomposite, the cellular uptake efficiency of CoHCF, CoHCF@MWCNTf, and CoHCF@MWCNTf@FA was quantified in MDA-MB-231 cells using UV–vis spectroscopy. The obtained results, expressed as mean uptake percentage ± standard deviation, are presented in Table 1.

**Table 1:** % Uptake of Nanocomposites (0.5 mg/mL) in MDA-MB-231 Cells

nanocomposite	uptake (%) ± SD
CoHCF	68.01 ± 0.96
CoHCF@MWCNTf	60.82 ± 2.90
CoHCF@MWCNTf@FA	69.05 ± 3.78

These results confirm that all materials are efficiently internalized by tumor cells, with uptake values around 60–70%. Notably, the folic acid functionalization slightly increased the internalization efficiency compared to the nonfunctionalized system (CoHCF@MWCNTf), consistent with the presence of folate receptor-mediated endocytosis in triple-negative breast cancer cells. This enhanced uptake likely contributes to the higher phototoxic response observed for CoHCF@MWCNTf@FA under light exposure (Figure 6f), as improved internalization facilitates greater intracellular ROS generation and interaction with critical biomolecular targets.

These findings provide additional mechanistic support for the proposed synergistic effect among CoHCF (photosensitizer), MWCNTf (tumor penetration enhancer), and FA (targeting ligand), reinforcing that the biological activity of the ternary nanocomposite is not merely due to extracellular effects or cobalt leaching but to active intracellular accumulation followed by light-triggered photodynamic action.

## Conclusions

In summary, this study reported the synthesis of a new nanocomposite through the coprecipitation method as a material to enhance PDT activity. SEM characterization showed an increase in the average particle size when the materials were combined, along with the cubic morphology for the materials. EDS measurement presented all the corresponding elements, while XRD indicated peaks mainly related to the face-centered cubic structure of the metal hexacyanoferrates, with a presence of rhombohedral distortion when MWCNTf was included. TGA analysis revealed that the material contains 45% water in its structure, a phenomenon that can be better evaluated by Raman and FTIR spectra, which showed the respective bands for the nanocomposites. Additionally, the voltammograms provided information on the oxidation states of CoHCF and its nanocomposites. UV–vis and diffuse reflectance spectra confirmed an absorption band in the visible range, potentially useful for tumor treatment by PDT. The stability studies in DMEM demonstrated that all nanocomposites maintained their characteristic absorption bands and structural integrity over 24 h, indicating good dispersion, minimal cobalt leaching, and overall stability under physiological-like conditions. Furthermore, cellular uptake assays confirmed efficient internalization of all nanocomposites by tumor cells, with uptake values around 60–70%. The FA incorporation slightly increased the internalization efficiency compared to the CoHCF@MWCNTf system, consistent with receptor-mediated endocytosis in triple-negative breast cancer cells. CoHCF@MWCNTf@FA exhibited a pronounced photodynamic effect at concentrations of 0.8 and 1.0 mg mL^–1^. The best condition for PDT against the tumor cell line found in this work was treatment with the CoHCF@MWCNTf@FA nanocomposite under red LED irradiation, which resulted in 79% cell death. Our data also suggest that the ternary nanocomposite induces early apoptosis in human breast cancer cells. Moreover, a clear selective cytotoxic profile was observed: upon irradiation, MCF-7 cells exhibited a marked, concentration-dependent reduction in viability, with values dropping below 44% at concentrations of 0.8 and 1.0 mg mL^–1^. In contrast, MCF-10A nontumorigenic breast epithelial cells maintained viability above 84% across all tested concentrations, regardless of light exposure, indicating low toxicity in healthy cells. Overall, this present work demonstrates that the synthesized CoHCF@MWCNTf@FA nanocomposite is a promising photosensitizing agent for the treatment of breast cancer, particularly triple-negative breast cancer (TNBC), a highly aggressive and therapeutically challenging subtype. Its ability to selectively induce cell death in tumor cells, while preserving the viability of healthy breast epithelial cells, highlights its potential for targeted and minimally invasive PDT. These findings reinforce the relevance of nanostructured systems in advancing more effective and safer cancer treatment strategies.

## Supplementary Material



## Notes

Parameters of the laser used in this study, EDS spectra of the materials, XRD data highlighting peak splitting in the CoHCF@MWCNTs@FA nanocomposite, absorption band frequencies observed in the FTIR spectra emphasizing the region corresponding to the ν­(C≡N) stretching, thermal decomposition data derived from TGA analysis, and UV–vis absorption spectra of nanocomposites dispersed in DMEM over 24 h of incubation (PDF)
