# Supplementary material for: Synergistic Effects of CoHCF@MWCNTs@FA Nanocomposites Enhancing Photodynamic Therapy for Triple-Negative Breast Cancer
Source: ACS Omega. 2026 Mar 12;11(11):17124–36. doi: 10.1021/acsomega.5c04309 (PMC13019244; doi:10.1021/acsomega.5c04309)
Supplement: Supplementary file 1 [file ao5c04309_si_001.pdf]

Synergistic effects of CoHCF@MWCNTs@FA nanocomposites enhancing photodynamic therapy for triple-negative breast cancer

Hellen C. Novais de Oliveira[a], *Patrícia A. Matos*[a], *Tayana M. Tsubone*[a], *and Edson Nossol*[a,*]

[a] Institute of Chemistry, Federal University of Uberlândia, Uberlândia, MG 38400-902, Brazil

Supporting information

Table S1: The parameters of the laser used in this study.

Parameter	Continuous wave laser
**Wavelength**	630 nm
**Wave emission**	Continuous
**Output power**	4.6 mW
**Duration of irradiation**	900 s
**Distance between laser and samples**	7 cm
**Sensor area**	0.785 cm^2
**Energy density**	5 J cm^{-2}

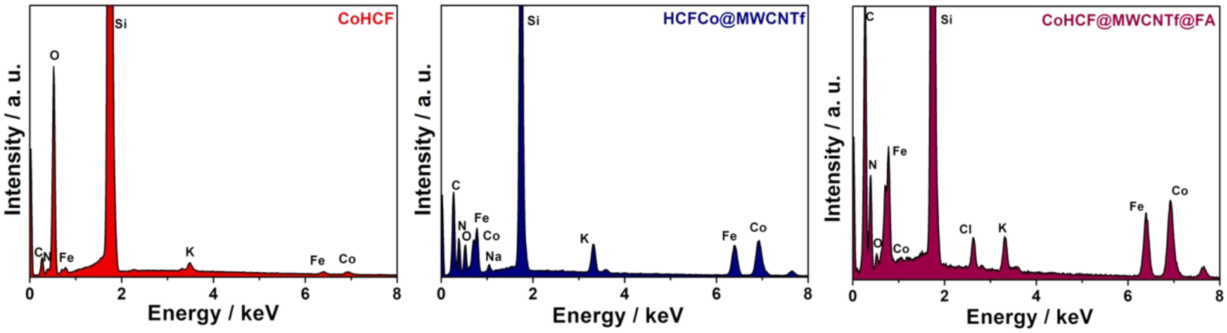

Figure S1: EDS spectra of CoHCF, CoHCF@MWCNTf and CoHCF@MWCNTf@FA.

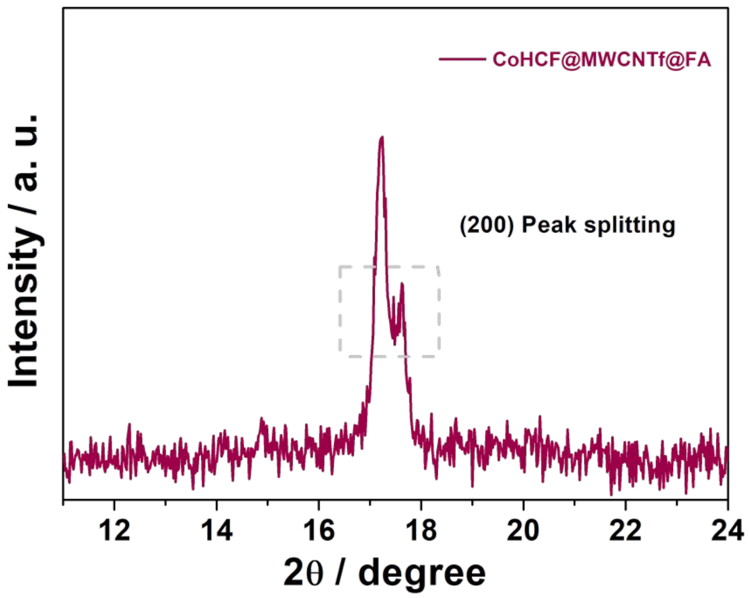

Figure S2: XRD peak splitting at 17,7 °for CoHCF@MWCNTf@FA.

Table S2: Frequency (in cm⁻¹) of the absorption bands observed for the study of the synthesized nanocomposites and their controls.

Sample	υ(CN)	δ(MCN)	υ(MC)	υ(OH)	δ(HOH)	ν(NH)	ν(CO)	ν(CH)
CoHCF	2093	590-537	422	3395	1606	-	-	-
CoHCF MWCNTf	2093	590-537	422	3395	1606	-	-	-
CoHCF MWCNTf FA	2084	590-537	422	3360	1606	3321	1690	1412

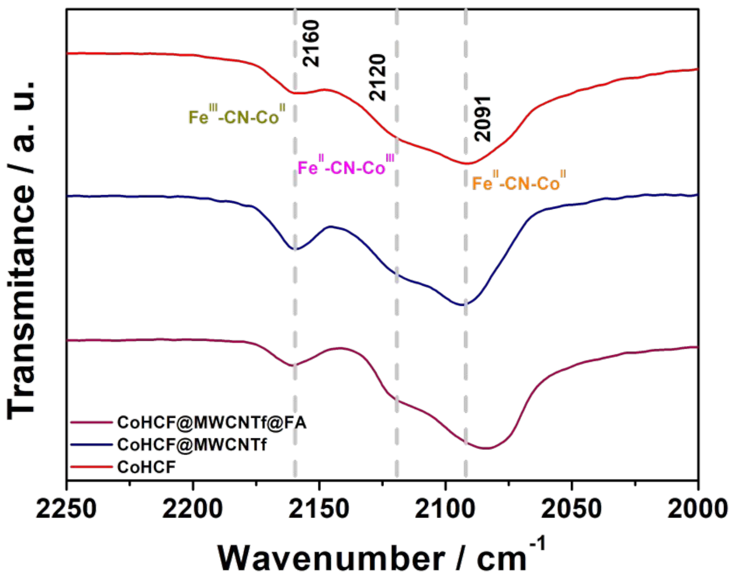

Figure S3: Highlighted region corresponding to the υ(C≡N) stretching vibration (2000–2500 cm^{-1}).

Table S3: TGA: thermal decomposition.

Materials	1ª decomposition		2ª decomposition		3ª decomposition	
	% weight	dTGA $_{max}$	% weight	dTGA $_{max}$	% weight	dTGA $_{max}$
CoHCF	23,33	122 °C	10,02	270 °C	14,48	302 °C
CoHCF@MWCNTf	20,58	119 °C	8,51	272 °C	15,79	302 °C
CoHCF@MWCNTf@FA	21,04	125 °C	9,38	278 °C	16,68	295 °C

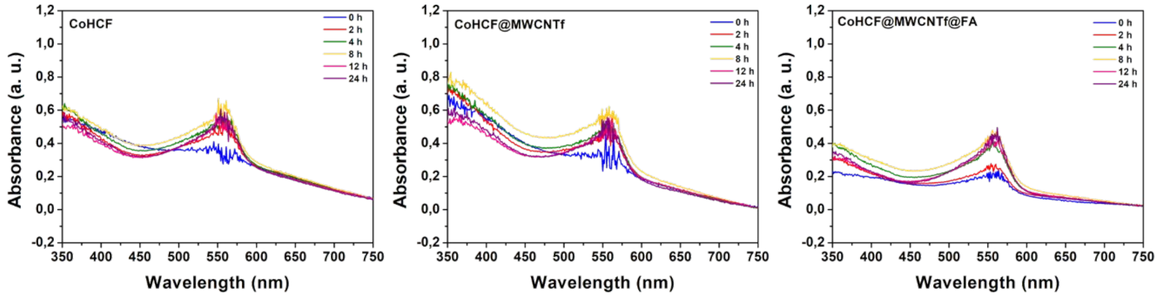

Figure S4: UV–Vis absorption spectra of CoHCF, CoHCF@MWCNTf, and CoHCF@MWCNTf@FA nanocomposites dispersed in DMEM over 24 h of incubation.
